# Cysts of the Broad Ligament of the Uterus

**Published:** 1879-05

**Authors:** R. Stansbury Sutton

**Affiliations:** Pittsburgh, Pa.


### ARTICLE II.

CYSTS OF THE BROAD LIGAMENT OF THE UTERUS. Notes of Three Cases by R. STANSBURY SUTTON, A.M., M.D., of Pittsburgh, Pa.

During the last few years I have met with three cases of cysts in the broad ligament. These cases all differed from one another in these particulars: Case I, the cyst was very large, and had frequently been tapped; Case II, the cyst was of medium size and had never before been diagnosed; Case III, the cysts were concentric, multiple and small, and, so far as my experience goes, the case is unique. Before relating these cases, I may be pardoned for making a few remarks upon the subject in general. Dr. Charles Clay seems to have been the earliest observer of these larger cysts. The general pathologist says very little about them, and as is remarked by Dr. Peaslee, the "ovariotomists in this country and in England" are those who have recognized them. Prior to 1850 Dr. Clay seems to have been about the only one who spoke of them. Now that ovariotomy is an established operation, we may expect to learn more of these cysts, which will be met with, whether we will it or not. Two of the cases I am about to report, may be put under the head of "large serous cysts of the broad ligament;" they are of slow growth and affect the general health very slowly. We are taught that after tapping they rarely fill again. In this particular my cases are at variance with the rule, as will be seen, but out of 40 cases Dr. Charles Clay cured 34 with the trocar, therefore two cases taken alone mean nothing; but if some one, collating such cases, will add these two to his number, that statistics may, after a while, be made up, for our own country, I will feel amply repaid for my efforts to place these cases within his reach.

*Case I.*—Miss Frances Husack, a resident of Pittsburgh, aged 21 years, presented herself at my office July 7, 1876. Her parents were Bohemians. She was born in Pennsylvania and had, living and well, two sisters and three brothers. Until five years ago, or up to her 16th year, her health was excellent. She menstruated for the first time at 14. At 16 she became irregular, and observed, about this time, an increase in the size of her abdomen. A year after this she was tapped by Dr. John Dickson, and eight liters of clear fluid were removed. She describes the fluid as having been "clear as well water." Thirteen months later she was tapped again by the late Dr. A. G. Walter, and ten (10) liters of similar fluid were removed. Eight months later she was tapped again by Dr. Walter, and 12 liters of similar fluid were removed. On the 10th of August, 1875, she was subjected to the fourth tapping, this time by Dr. Duff; 16 liters of clear fluid were removed. It is therefore scarcely a year to-day since she was tapped. She reports a good appetite, daily action of the bowels, and the ability to sleep well.

*Measurements.*

| | |
|---|---|
| Around the body at the umbilicus........................ | 106 Cm. |
| From ensiform cartilage to the umbilicus................ | 23 " |
| From pubis to the umbilicus.............................. | 27 " |
| From R't super. spinous process of ilium to umbilicus, | 26½ " |
| From L't " " " " " " | 27 " |
| Around the body, 5 Cm. above the umbilicus.......... | 104 " |

Fluctuation is very distinct over the entire swelling. Change of position does not alter the boundaries of fluctuation. The abdominal walls glide readily under the hand over the anterior and lateral aspect of the growth. The epigastric veins are slightly enlarged. The umbilicus is level with the general surface. The patient is considerably emaciated. The expression of countenance is not decidedly ovarian. The uterus is retroverted, and measures in depth 8½ Cm. The following facts gained in this examination may be tabulated:

1. The patient is young, viz., 21.

2. The growth has not been rapid.

3. The tumor is large, but the general health tolerably good.

4. The abdominal veins are less enlarged than usual in great abdominal distensions.

5. Fluctuation is very distinct and continuous over the entire growth.

6. The expression of countenance is not decidedly ovarian.

July 31 (24 days since first examination), I introduced a large aspirator needle into the cyst, and through it withdrew 17 liters of fluid literally as "clear as well water." This fluid possessed high refractive power, contained no albumen, but did contain chloride of sodium. Its specific gravity was 1008. It was very apparent that the cyst was one of the broad ligament. At this tapping the cyst was not emptied, but considerable fluid was intentionally left in it. I ordered tincture ferri chloridi 1 Gm. 35 centigrams, *ter die*, and good diet. Also lithia citrate, 35 centigrams in a glass of water each morning before eating.

August 14.—At 10:30 a.m., Squibb's ether was administered by Dr. Giesking. Assisted by Dr. Rohauser, Dr. Purviance and Dr. Lemoyne, I proceeded to operate. An incision 10 Cm. long was carried through the abdominal wall in the line of the linea alba. The cyst, partially empty, was seized with a tenaculum and partly drawn through the incision. Fitch's trocar was now introduced, and the cyst rapidly emptied of 6 liters of fluid. It was now carefully drawn out, and found to be a cyst of the left broad ligament. Its pedicle was very short and very broad. The ovary adherent to the cyst was healthy. A needle, threaded with four strands of carbolized silk, was now carried through the pedicle about at its center. The needle was cut away and each half of the pedicle was firmly tied with two strands of the ligature. The pedicle was now severed $2\frac{1}{2}$ Cm. beyond the ligature, and the cyst removed. After sponging out the belly, the ligatures were cut off close to the knots and the pedicle dropped in. The wound in the abdominal wall was now closed with carbolized silk sutures and adhesive straps; a bandage being adjusted, the patient was put to bed; pulse 31. Pain being complained of, an injection of 1 centigram of morphia was given subcutaneously, a jug of hot water was placed at the feet, and milk and lime water ordered to satisfy either thirst or hunger. At 10 p. m., temperature 38.4, pulse 41.2.

Aug. 15, 10 p.m.—Temperature 38.3, pulse 41.2. Removed two stitches from the wound.

Aug. 16, 10 p.m.—Temperature 37.9, pulse 40. Removed two stitches from the wound.

Aug. 17, 10 p.m.—Temperature 38.2, pulse 46.6. Removed the last stitch ; wound closed.

Aug. 18, 10 p.m.—Temperature 38, pulse 42. Is eating well ; ordered quinia sulphate 35 centigrams and tincture opii gtt. x per rectum daily as a tonic and to avoid tympany.

Aug. 19, 10 p.m.—Temperature 38.2, pulse 36.7. Taking also to-day a dessert spoonful of brandy at meal time.

Aug. 20, 10 p.m.—Temperature 37.5, pulse 37.8.

Aug. 21, 10 p.m.—Temperature 37.5, pulse 37.8. Complains of frequent desire to pass fæces, without any result. On passing a finger into the rectum, it was found packed. The pack was broken up, and with the aid of injections of soap suds the rectum was emptied of a large quantity of fæcal matter.

Aug. 22.—Temperature 37.3, pulse 28.9.

Aug. 24—Temperature 37, pulse 26.7. Sitting up in bed.

Aug. 30—Temperature 37, pulse 26.7. Sitting up, out of bed ; appetite good.

Sept. 2.—Slight intercostal neuralgia ; ordered a flannel jacket.

Sept. 3, .—Neuralgia gone.

Sept. 4.—Patient discharged.

During the first four days after the operation, the catheter was used every six hours by Dr. Rohauser, who lived but a few doors from the patient, and whom I retained in the case with me. At this date, May 25, 1878, this patient is now a healthy married woman.

*Case II.*—On the 17th of March, 1877, I was requested by my friend, Dr. Joseph Dickson, of this city, to see with him a Mrs. ——, from an adjoining county. She was a thin, tall woman of a highly nervous temperament. She was 34 years of age, had married at 15, and enjoyed fair health for about ten years afterwards. She had never borne children, which fact was possibly due to the existence, until recently, of a long, conical cervix, with a narrow canal. Her menstruation had always been painful. She had consulted many physicians during the last nine years. Her symptoms were peculiar. She was painfully nervous and irritable. As she stood before us, she placed her hands upon the lateral walls of the abdomen, and by means of pressure, first on one side, then upon the other, produced a rumbling, bubbling noise in the intestines, which was a surprise to us both. Some time before, she said, the neck of the womb had protruded between the labia and had been amputed. Since the operation she had menstruated more comfortably, but her general nervous condition was no better, and she had threatened to destroy herself. We decided to carefully examine the abdominal and pelvic viscera. Percussion was first in order. Clear sounds were elicited all along the entire course of the colon, or more properly throughout the right inguinal, right lumbar, right hypogastric, epigastric, left hypogastric, left lumbar, and nearly all of the left inguinal and umbilical regions.

At a point $2\frac{1}{2}$ Ctm. below the umbilicus, a dull sound was found; this dullness extended down to the pubes, and corresponded with the umbilical region in width, overlapping the line a little on the left side. Indistinct fluctuation was evident. The region occupied is that which a full bladder would fill. A catheter passed proved the bladder to be empty, and the catheter could be felt through the anterior wall of the vagina as high as the neck of the uterus. Two fingers passed into the vagina, and the other hand pressed down above the pubic symphysis, fairly caught the cyst, and gave a tolerably accurate idea of its size.

The uterus was pressed back upon the rectum, and measured 6 Ctm. with the sound. We concluded that the patient had a cyst either of the broad ligament or Fallopian tube, and I requested Dr. Dickson to aspirate it. The belly was in appearance perfectly normal.

Returning to the room in which the patient lay, Dr. Dickson thrust an aspirator needle into the cyst, and drew off 1 liter, 24 C. C. of clear fluid, of a low specific gravity, free from albumen but rich in chloride of sodium, and possessing high refractive power.

I left the patient to the Doctor, since which time I have not examined her. On inquiry, Dr. Dickson tells me that the cyst refilled, and he has tapped it once since. The nervous symptoms continue.

*Case III.*—On Dec. 7, 1877, I saw, in consultation with Dr. Thomas Mahon, of Allegheny City, Pa., Miss Lizzie M., aged 15 years, sent to him by Dr. Hughes, of Blairsville, Pa. Her face was that of one apparently much older. She had never menstruated. Eighteen months ago she was perfectly well. Soon after this date she observed her abdomen growing larger, and also suffered some pain in the left side. Six months later her abdomen had become very large. Passing along a pathway in the yard, *she fell over a water-bucket.* She was not apparently injured. The following day she observed *a change in her shape*, and the swelling of her abdomen *rapidly disappeared.* She says *no fluid* escaped by the *vagina.* About three months after this the abdomen again began to enlarge, and at the end of nine months, viz., in October, 1877, Dr. Hughes tapped her of many liters of fluid, greenish in color, thick and heavy. Two months later, viz., Dec. 2, 1877, he again tapped her of 19 liters 200 C. C. of similar fluid. Such is the history given by herself and her father, who accompanies her. It is, therefore, only five days since she was tapped. This fluid was not examined with the microscope. By palpation I detect considerable fluid in the belly at this time ; its presence is confirmed by gravity carrying it about on change of position, dullness on percussion locating it. But between two fingers in the vagina and the hand in the left inguinal region, I feel what I take for a cyst nearly empty ; it is soft and elastic. The upper hand detects a round body, which I suspect to be a solid body in it. The patient is so restless, however, that we desist. Relying upon the history and what we have found, we conclude that we have to deal with an ovarian cyst. Three weeks after this I met Dr. Hughes in Blairsville, and visited this patient with him. She was very large, and I wanted some of the fluid. Dr. Hughes tapped her of 9 liters 500 C. C., leaving a great deal in her. This fluid was greenish in color, sticky and heavy. It contained albumen. Specific gravity 1015. At this visit I did not *re-examine the case*—in my opinion, a great mistake. I brought home a bottle of the fluid, and examined it with the microscope. It contained epithelium and a granular cell which I took to be the ovarian cell. I sent to each of two good microscopists in the city a specimen of the fluid. They were ignorant of the origin of the fluid. After examining it they pronounced it to be fluid from an ovarian cyst. This impartial and independent confirmation of our diagnosis put my mind at rest, and at the request of Drs. Hughes and Mahon I decided to remove the cyst when they considered the patient ready.

On the 15th of January, 1878, at the patient's home, I met Dr. Hughes and a number of medical gentlemen of Blairsville.

I made an opening in the linea alba 6 Ctm. long. No ovarian cyst was to be seen. An enormous quantity of fluid was removed from the belly. The small intestines were glued in a lump, and lay high up in the abdomen. The vessels of the peritoneum were congested; the ovaries and uterus were healthy, but between the layer of the left broad ligament there was something filling up the pelvis. I cleaned the fluid out of abdomen, announced a mistake in diagnosis, and closed the wound. Four days afterwards I received a postal from Dr. Hughes saying the patient was dead, and that he had removed at the post-mortem the uterus and ovaries.

Two days afterwards I examined the uterus, broad ligaments, etc., with Dr. Hughes and several of the physicians of Blairsville. The uterus and both ovaries were healthy, but between the layers of the left broad ligament there was a soft elastic mass as large as a California or Florida orange. The entire mass lay below or under the ovary and tube, and was covered by the serous layers of the broad ligament. Between the mass and the uterus there was 1¼ Cm. of the ligament unsplit by the mass. In the serous covering was found a small opening communicating with the mass; out of this opening oozed a glassy, amber colored fluid, when the specimen was pressed upon. The serous covering was now slit open, revealing a mass of hydatiform cysts, varying in size from a pea to a shell-bark hickory nut. Many of these cysts were concentric, their walls were transparent and exceedingly thin. The contents of the cysts was a fluid heavier than water, in color resembling currant jelly. Microscopic examination failed to show any hooklets. The case was one of chronic peritonitis, complicated by this mass of cysts. Did this mass of cysts cause the peritonitis ? On account of professional engagements, Dr. Mahon was not present at the operation.

